# Mechanism of Activation and Mechanical Properties of Alkali-Activated Material Derived from GGBFS/FA Activated by Carbide Slag

**DOI:** 10.3390/ma19071313

**Published:** 2026-03-26

**Authors:** Zhong Wang, Shujie Chen, Xiaoyan Zheng, Xia Huang, Tengfei Fu, Chao Feng, Demei Yu, Hengchun Zhang

**Affiliations:** 1 Fuzhou Luxin Highway Design Co., Ltd., Fuzhou 350007, China; 2 College of Transportation and Civil Engineering, Fujian Agriculture and Forestry University, Fuzhou 350108, China; 5221342021@fafu.edu.cn (S.C.); xiaoyanzheng@fafu.edu.cn (X.Z.); futengfei@fafu.edu.cn (T.F.); yudemei0826@fafu.edu.cn (D.Y.); 3 CSCEC Strait Construction and Development Co., Ltd., Fuzhou 350015, China

**Keywords:** alkali-activated material, workability, strength, hydration characteristics, microstructure, activating mechanism

## Abstract

Ground granulated blast furnace slag (GGBFS)-based cementitious materials, known for their high strength and good fluidity, present an eco-friendly, low-carbon alternative to ordinary Portland cement (OPC). However, the high cost of activators poses a significant challenge, accounting for over 50% of alkali-activated material production costs. This study uses carbide slag (CS), a byproduct of polyvinylchloride (PVC) production, as an activator, along with other solid wastes such as GGBFS and fly ash (FA) as precursors to develop a novel, low-carbon alkali-activated material binder made entirely from solid waste. Various mixtures with different proportions of CS and GGBFS were prepared, and their workability and strength were tested at different ages. Additionally, the hydration characteristics and microstructure of the samples were analyzed using XRD, TG-DTG, FTIR, heat of hydration tests, and SEM-EDS. Results show that calcium hydroxide in CS activates the pozzolanic activity of GGBFS and FA, improving the strength as the proportion of CS increases. At the 5% CS content, the 7 days compressive strength of the GGBFS-based alkali-activated material increased by 79.7% compared to a 2% CS content. However, adding CS reduces the workability of the polymer slurry, with a spread decrease of 168.5 mm and 161.5 mm as the CS content increases from 2% to 8%. The inclusion of CS also increases the rate and total heat released during hydration, with the optimal performance observed at 5% CS. While FA incorporation reduces strength, it enhances slurry workability and reduces heat release during hydration. The strength development is attributed to the formation of AFt, C-S-H gel, C-(A)-S-H gel, and hydrocalumite-like hydrates.

## 1. Introduction

The ordinary Portland cement (OPC) industry accounts for 5% to 7% of global CO_2_ emissions. To reduce the carbon footprint of OPC production, it is crucial to develop alternative binders that emit less CO_2_ [1,2]. Alkali-activated material, formed by the geo-polymerization of silicate and aluminate tetrahedra from alkali-activated natural minerals or solid wastes rich in aluminosilicates (e.g., GGBFS [3] and FA [4]), offers a promising solution. Unlike OPC production, alkali-activated material production does not require calcination, which significantly reduces energy consumption and CO_2_ emissions. It also allows the use of industrial solid wastes and abundant, low-cost raw materials [5].

The chemical composition of GGBFS primarily includes SiO_2_, CaO, and Al_2_O_3_ [6]. Numerous studies have shown that GGBFS can effectively replace OPC [7,8], significantly reducing energy consumption and carbon emissions [9,10] during its production. FA, a pozzolanic waste, is composed mainly of oxides such as SiO_2_, Al_2_O_3_, and Fe_2_O_3_ [11]. Improper storage of FA requires vast amounts of land and can disrupt soil pH balance. CS, consisting of Ca(OH)_2_, CaCO_3_, and a small amount of silicates, has a high pH (>12) and strong alkalinity in aqueous environments, providing a sufficient calcium source for activation. With an annual production of over 28 million tons in China alone, CS shows great potential as a green alkaline activator [12,13]. Previous studies have demonstrated the feasibility of using CS to activate GGBFS-based alkali-activated material. Li et al. found that CS can replace slaked lime to activate GGBFS-based alkali-activated material [14], and Gao et al. showed that CS can enhance the early strength of Na_2_CO_3_-activated GGBFS-based alkali-activated material [15]. Zhao et al. used CS to prepare a mixed alkali activator and found that it mitigated the rapid hardening and exothermic reactions in alkali-activated material [16]. Microscopic characterization studies have confirmed that the incorporation of CS effectively induces the formation of C-S-H and C-(A)-S-H gels with dense microstructures, which highlights its promising potential as an eco-friendly activator in geo-polymerization reactions for cementless composite development [17,18,19,20].

Alkali-activated material, formed through the reaction of aluminosilicate precursors with alkaline activators [21,22,23], exhibits mechanical and durability properties similar to OPC. However, alkali-activated material, especially that made from GGBFS, is challenging to process due to its short setting times and significant volume shrinkage during hardening, which can reduce its mechanical performance. Alkali-activated material made primarily from FA exhibits good workability but faces challenges in strength development. To overcome these issues, researchers have combined GGBFS and FA to create alkali-activated composite binders that combine the strengths of both materials [24]. While previous research has explored the microstructure and hydration products of mineral-based alkali-activated material [25,26,27,28,29], establishing a clear link between macroscopic properties and microstructure remains challenging. Thus, further research on the workability, mechanical properties, and hydration characteristics of alkali-activated materials is necessary to establish a comprehensive understanding of their performance.

The research examines the workability and strength of CS-activated GGBFS-based and GGBFS-FA (GFA)-based alkali-activated materials. Analytical techniques such as X-ray diffraction (XRD), Thermogravimetry-differential thermogravimetry (TG-DTG), Fourier-transform infrared spectroscopy (FTIR), Scanning electron microscopy-Energy dispersive spectroscopy (SEM-EDS), and heat of hydration were employed to characterize reaction products and elucidate the activation mechanisms of GGBFS and FA. Findings from this research have broadened the applications of CS as an alkaline activator, providing insights into the relationship between material microstructure and mechanical properties. The findings provide an innovative strategy for alkali-activated material formulation, facilitating the advancement and practical implementation of GGBFS and GFA-based low-carbon cementitious composites.

## 2. Materials and Testing Methods

### 2.1. Materials

**Figure 1:**
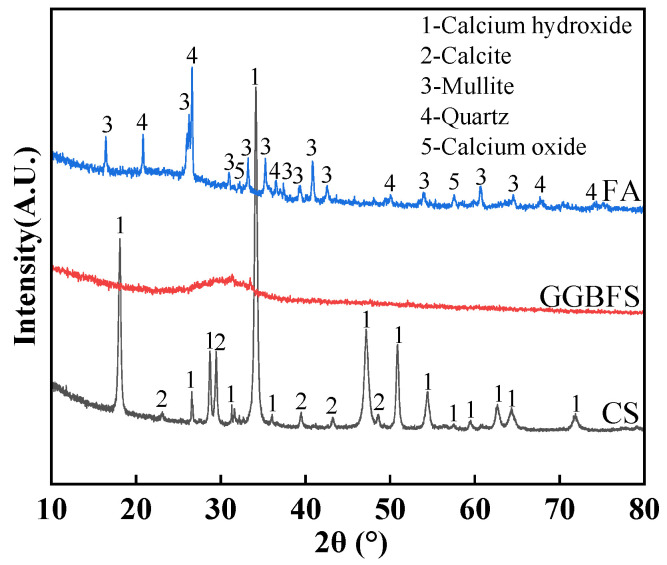
Phase composition of materials.

**Figure 2:**
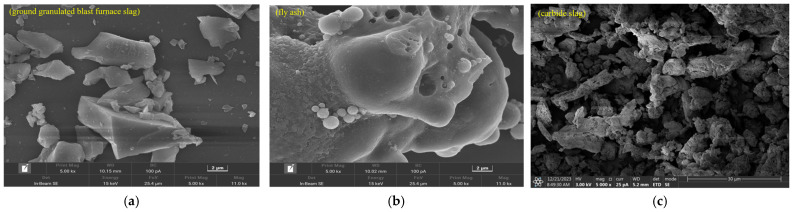
SEM images of materials: (**a**) GGBFS (2 μm scale bar), (**b**) FA (2 μm scale bar), and (**c**) CS (30 μm scale bar).

The materials include GGBFS, FA, and CS. GGBFS is the grade S95 granulated blast-furnace slag, produced by Henan Wuhu Environmental Protection Technology Co., Ltd. (Zhengzhou, China), with a density of 3.10 g/cm^3^ and a specific surface area of 430 m^2^/kg. FA meets the technical requirements for Class F fly ash and is produced by Fujian Huadian Kemen Power Generation Co., Ltd. (Fuzhou, China). CS is a solid waste generated from the calcium carbide-based acetylene production process in a polyvinyl chloride (PVC) manufacturing plant, sourced from Henan Wuhu Environmental Protection Technology Co., Ltd. (Zhengzhou, China). It exhibits a strong alkalinity with a measured pH value of 12.7. The water reducing agent is a polycarboxylate-based with a water reduction rate of 27.5%. The GGBFS, FA, and CS were sieved through a 75 µm sieve and then sealed for subsequent material property tests. The chemical compositions and phase analysis results of GGBFS, FA, and CS are presented in Table 1 and Figure 1, which were obtained by J6 JAGUAR X-ray fluorescence spectrometer (Bruker, Karlsruhe, Germany) and X’Pert PRO X-ray diffractometer (Malvern Panalytical, Almelo, The Netherlands), respectively. SEM imaging reveals that GGBFS and CS particles exhibit irregular shapes with distinct edges and corners, while the irregular non-spherical FA morphology is caused by fusion adhesion of high-viscosity molten droplets combined with surface collapse and pore formation due to gas escape during cooling (Figure 2).

### 2.2. Mixture Design

The mixture design for this experiment is outlined in Table 2. The water-to-binder (W/B) ratio was set to 0.34, and the mass ratios of GGBFS to FA are varied as follows: 100/0, 70/30, 50/50, 30/70, and 0/100. CS was added at varying amounts (2%, 3%, 4%, 5%, 6%, 7%, and 8% by the total mass of GGBFS and FA). The mass of mixing water was measured accordingly, and a water-reducing agent was added at 0.5% of the total mass of the cementitious material to enhance fluidity.

After stirring the mixture to form a homogeneous paste, 40 mm × 40 mm × 160 mm prismatic specimens were fabricated, which were then sealed with plastic film for moisture retention, with no fewer than three replicates prepared per mixture. All specimens were initially cured at room temperature for 24 h prior to demolding; post-demolding, they were transferred to a standard curing chamber (temperature: 20 ± 2 °C, relative humidity: >95%) and maintained until the target testing age.

### 2.3. Test Methods

The flowability of the fresh paste was tested following GB/T 8007-2012 [30]. The paste was filled into a frustum-shaped mold (upper diameter 36 mm, lower diameter 60 mm, height 60 mm) and permitted to spread freely on a glass plate for 30 s. The maximum spread diameter of the paste was measured in two perpendicular directions, with the average value taken as the final flowability index.

The strengths of hardened specimens were determined in line with GB/T 17671-2020 [31], using a CDT 1305-2 microcomputer-controlled electronic universal testing machine (MTS, Shanghai, China). For the flexural strength test of each mixture, three specimens were tested at a loading rate of 50 N/s ± 10 N/s, while six specimens were tested for compressive strength at a loading rate of 2400 N/s ± 200 N/s.

### 2.4. Techniques for Material Characterization

Before analysis, specimens were broken into smaller pieces, soaked in anhydrous ethanol for 72 h to terminate hydration, and then dried at 50 °C to a constant weight. A portion of the crushed specimens was ground into a fine powder using an agate mortar and pestle, then passed through a 200-mesh sieve for XRD, TG-DTG and FTIR analysis. Samples for SEM and EDS were taken from the center of the dried specimens (1 cm × 1 cm).

The hydration exothermic rate and cumulative heat release of different alkali-activated material systems were determined with a TAM Air isothermal calorimeter (TA Instruments, New Castle, DE, USA), following ASTM C1702-2023 [32]. The W/B ratio was maintained at 0.34, with two replicates tested per mixture and the results averaged for reliability.

The phase composition of the materials and alkali-activated material samples was analyzed using an X’Pert PRO X-ray diffractometer from The Netherlands. The scanning range for diffraction angles (2θ) was from 10° to 80°, with a scanning speed of 5°/min. Analysis was conducted at ages of 3 days, 7 days, and 28 days.

TG-DTG analysis was conducted using a NETZSCH STA 449F5 instrument (NETZSCH, Hanau, Germany). In a nitrogen atmosphere, they were heated at a rate of 10 °C/min from 30 °C to 1000 °C. The changes in mass caused by the heat-induced endothermic or exothermic reactions of the tested samples were observed, and the entire curve during the mass change process was recorded. The percentage of the weight loss portion in the entire sample was calculated.

The analysis was conducted using the Nicolet IS10 infrared spectrometer (Thermo Fisher, Waltham, MA, USA). FTIR spectra were collected in the mid-infrared region of 4000–400 cm^−1^ with a resolution of 4 cm^−1^ and a scanning number of 32 times.

SEM and EDS point scanning analysis were performed on alkali-activated material samples at different curing ages using the TESCAN MIRA scanning electron microscope (Tescan, Shanghai, China).

## 3. Results and Discussions

### 3.1. Workability

**Figure 3:**
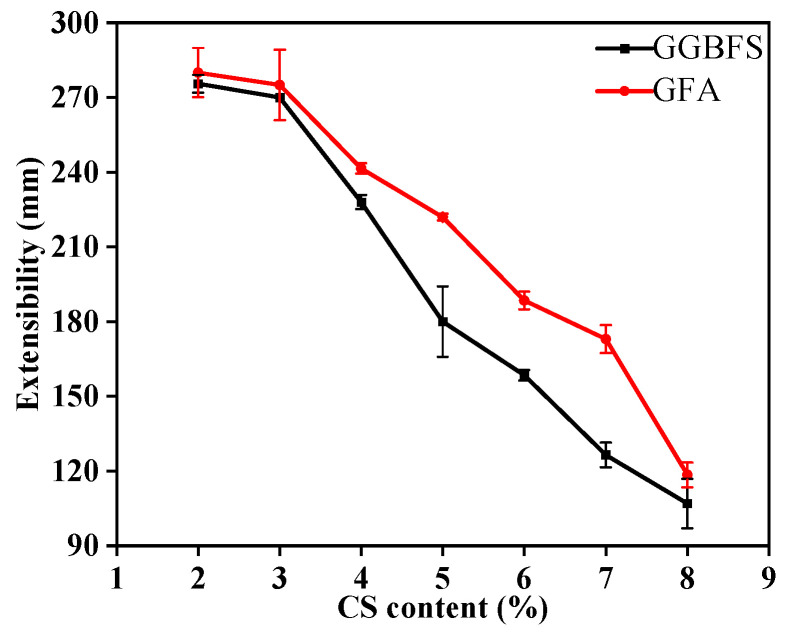
The influence of cs content on the extensibility of slurry.

The spread of the alkali-activated material paste decreases as the CS content increases (Figure 3). When the CS content increases from 2% to 8%, the spread of the GGBFS-based alkali-activated material paste decreases by 168.5 mm, and the GFA-based paste (GGBFS/FA = 70/30) decreases by 161.5 mm, respectively.

The addition of CS significantly impacts the flowability of the alkali-activated material paste, which can be attributed to two main factors: (1) As CS content increases, the water demand rises substantially. This is due to the increased solid phase content, which necessitates more water to maintain adequate flowability. The result is a higher paste viscosity, reducing its overall spread and flowability; (2) CS exhibits strong alkalinity, and as its content increases, the pH of the paste rises. In a highly alkaline environment, the reactivity of GGBFS and FA is activated, leading to the formation of flocculent hydration products and accelerating the coagulation and hardening process, further restricting the flowability of the paste.

### 3.2. Strengths

#### 3.2.1. Impact of CS Content

**Figure 4:**
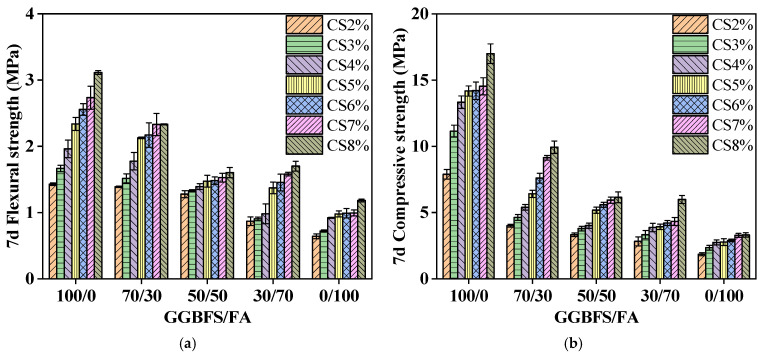
Strength curves of (**a**) flexural strength and (**b**) compressive strength specimens.

Figure 4 illustrates the effect of CS content on the strength of GGBFS-based and GFA-based alkali-activated material. As the FA content increases, the mechanical strength of the specimens tends to decrease. Additionally, higher FA content reduces the impact of CS on the strength.

For GGBFS-based alkali-activated material (GGBFS/FA = 100/0) at 7 days, the flexural strength varies by 1.69 MPa, and the compressive strength varies by 9.11 MPa between 2% and 8% CS content. For FA-based alkali-activated material (GGBFS/FA = 0/100), the flexural strength difference is 0.54 MPa, and the compressive strength difference is 1.45 MPa with varying CS content.

With the increase of CS content, the strength of alkali-activated material enhances. Specifically, with 5% CS, the 7 days flexural strength of GGBFS-based alkali-activated material reaches 2.3 MPa, and the compressive strength reaches 14.2 MPa, which represents an increase of 64.3% and 79.7%, respectively, compared with the 2% CS content. For GFA-based alkali-activated material (GGBFS/FA = 70/30), the 7 days flexural strength reaches 2.1 MPa, and the compressive strength reaches 6.4 MPa, showing increases of 50% and 60%, respectively, compared to a 2% CS content.

Within a certain range of CS content, the mechanical strength of alkali-activated material increases as CS content rises. This is attributed to the release of Ca(OH)_2_ from CS, which increases the concentration of Ca^2+^ and OH^−^ in the system. This promotes the geo-polymerization reaction, generating a substantial amount of geopolymer gels. The gel fills the internal pores of the alkali-activated material, making the matrix more compact and enhancing the strength of the material.

#### 3.2.2. Impact of GGBFS Content

**Figure 5:**
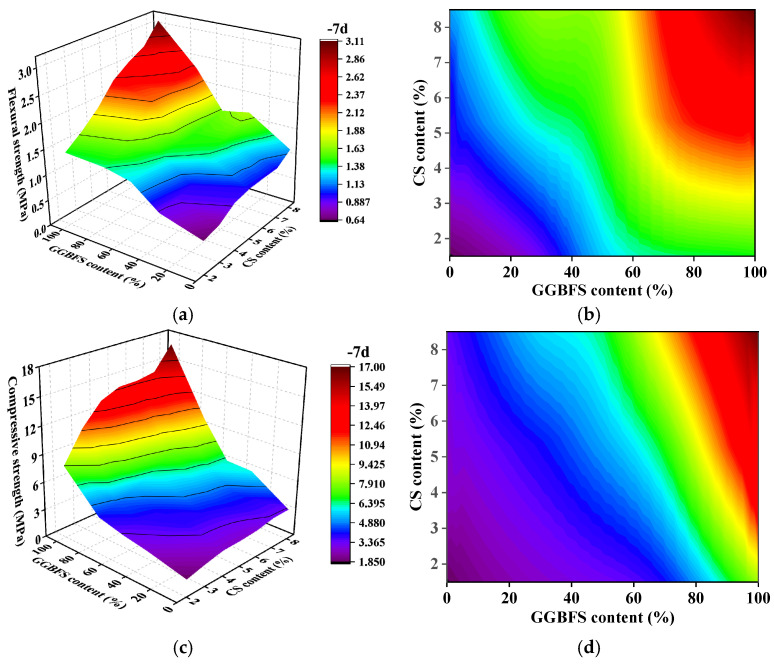
The influence of various factors on strengths: (**a**,**b**) flexural strength and (**c**,**d**) compressive strength.

The impact of the GGBFS-to-FA ratio (i.e., the GGBFS content) and CS content on the strength of alkali-activated material is shown in the contour plot (Figure 5). The strength of alkali-activated material increases with both GGBFS content and CS content. However, the impact of the GGBFS to FA ratio on strength is more pronounced than that of CS content.

When the CS content is fixed at 5%, the 7 days flexural strength of alkali-activated material varies from 2.3 MPa to 1.0 MPa as the GGBFS to FA ratio decreases. Alkali-activated material with 100% GGBFS content shows flexural strength more than 1.6 times higher than that with 30% GGBFS content. Similarly, the 7 days compressive strength ranges from 14.2 MPa to 3.9 MPa, with alkali-activated material containing 100% GGBFS showing more than 2.3 times the strength of those with 30% GGBFS content.

Furthermore, when the GGBFS to FA is held constant, increasing the CS content improves the compressive strength of alkali-activated material, but the effect is significantly smaller compared to the impact of the GGBFS to FA ratio. This shows that GGBFS content plays a dominant role in enhancing the strength of alkali-activated material.

This dominance can be explained by the higher reactivity of GGBFS compared to FA. When activated in an alkaline environment, GGBFS undergoes a hydration reaction, generating a significant amount of heat and causing a rapid rise in the internal temperature of the alkali-activated material. This promotes the geo-polymerization process, forming more cementitious material in a shorter time. While this heat effect is not sustained long-term, it is beneficial in the early stages of strength development, as it helps fill the pores between particles, improving the material’s integrity and strength. Additionally, the high CaO content in GGBFS leads to the formation of C-S-H gels and C-(A)-S-H gels as free Ca^2+^ reacts with Si and Al, which further enhances the mechanical strength. C-S-H can also act as a nucleation site, accelerating the formation of N-(A)-S-H gels and compacting the structure, thus increasing the mechanical properties of alkali-activated material.

#### 3.2.3. Impact of Curing Age

**Figure 6:**
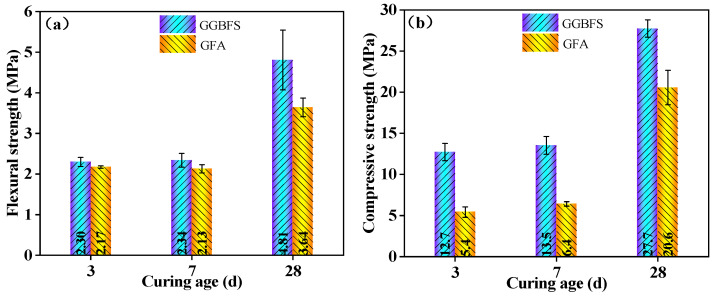
Strength development of specimens at 3 days, 7 days and 28 days. (**a**) Flexural strength. (**b**) Compressive strength.

As shown in Figure 6, the strength of alkali-activated material specimens increases steadily with curing age. At both 3 days and 28 days, the compressive strength of GGBFS-based alkali-activated material is consistently higher than that of GFA-based alkali-activated material (GGBFS/FA = 70/30), with differences of 7.3 MPa at 3 days and 7.1 MPa at 28 days, respectively. However, the flexural strength between the two types of alkali-activated materials at 3 days and 7 days does not show significant variation. The flexural strength is 2.30 MPa and 2.17 MPa at 3 days, and 2.34 MPa and 2.13 MPa, respectively, at 7 days for GGBFS-based and GFA-based alkali-activated materials, respectively.

The rate of strength gain accelerates in the later stages of curing, with the flexural strength at 28 d reaching 4.81 MPa for GGBFS-based alkali-activated material (a 106.0% increase from 7 days) and 3.64 MPa for GFA-based alkali-activated material (a 70.9% increase from 7 days). The compressive strength at 28 days also increases significantly, reaching 27.7 MPa for GGBFS-based alkali-activated material and 20.6 MPa for GFA-based alkali-activated material. The maximum 28 days compressive strength is 27.7 MPa, corresponding to concrete grade C20/25, which is suitable for non-structural applications such as non-load-bearing precast elements, road base materials, and backfill materials.

The early strength development in alkali-activated material is attributed to the high oxide content of Si and Al in the precursors, GGBFS and FA. In the initial stages of the reaction, Al oxides are the first to dissolve in the alkali-activated system, with the Al-O bonds in the glass body breaking. The [AlO_4_]^5−^ tetrahedra quickly dissolve, forming oligomers primarily based on Al-O-Al linkages. As the reaction progresses, Si oxides are gradually dissolved by CS, breaking the Si-O bonds and causing the [SiO_4_]^4−^ tetrahedra to dissolve. These then form oligomers connected by Si-O-Si linkages.

During the reaction, dissolved Si and Al oxides undergo polycondensation reactions at temperatures below 150 °C, forming calcium-containing hydrated gel phases that act as the primary binding matrix. These gel phases begin to precipitate and harden shortly after molding, contributing to the early-age strength development of the alkali-activated material. With continued curing, especially at 28 days, the oxide precursors in the high-calcium alkali-activated system are progressively dissolved. Further depolymerization and condensation reactions occur, leading to the formation of abundant layered C-S-H gel and C-(A)-S-H gel. The continuous growth and interlocking of these gel phases fill the pore structure, resulting in the peak strength observed in the specimens at 28 days.

### 3.3. Hydration Heat Analysis

**Figure 7:**
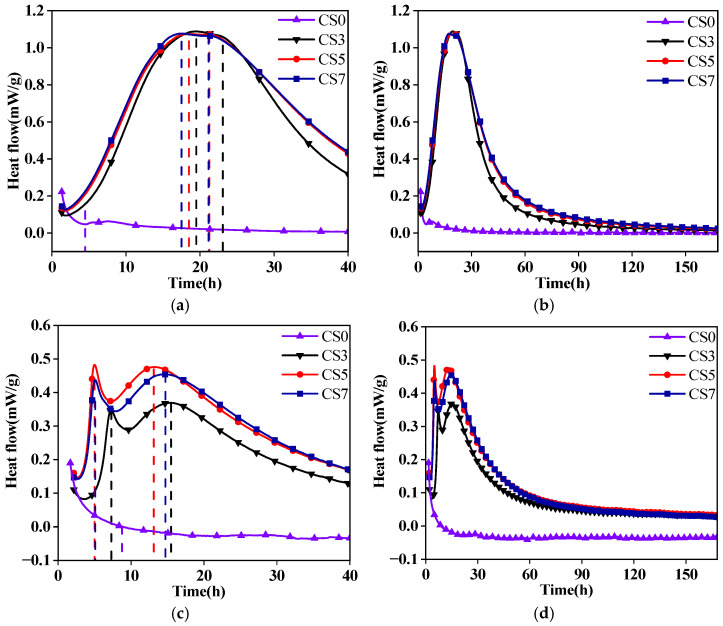
Heat release curves of GGBFS -based and GFA-based alkali-activated materials: (**a**,**c**) first 40 h and (**b**,**d**) 168 h.

Figure 7 shows the heat release curves of GGBFS-based alkali-activated material and GFA-based alkali-activated material (GGBFS/FA = 70/30). Figure 7a,b shows the hydration exotherm rate curves of GGBFS-based alkali-activated material with different CS contents. All curves reach their maximum exothermic rate between 17 and 20 h, with the peak occurring earlier as the CS content increases. The addition of CS rapidly activates the reactivity of GGBFS, leading to the formation of a significant amount of hydration products. However, when the CS content exceeds 3%, the impact on the hydration exotherm rate becomes minimal.

In Figure 7c, the hydration exotherm rate curves for GFA-based alkali-activated material with varying CS contents exhibit the first exothermic peak within 5 to 10 h. This peak appears the earliest and is highest when the CS content is 5%. The first exothermic peak results from the dissolution of silicoaluminate materials in an alkaline environment [33]. The increase and advancement of this peak indicate a more intense dissolution of these materials within the polymer system. At the CS content of 5%, more OH^−^ ions are supplied to the system, which, in turn, increases the release efficiency of silicoaluminate components in the GFA-based alkali-activated material. Figure 7d shows the heat release curve of the GFA-based alkali-activated material over a period of 168 h. It can be observed that after approximately 60 h, the heat flow rate of all CS-containing samples (CS3, CS5, and CS7) undergoes a rapid decay, gradually stabilizing at a very low level for the remainder of the testing period. This indicates that the dominant early-stage hydration/polycondensation reactions have been largely completed, and only residual reaction processes occur in the later stages.

Between 13 and 15 h, the hydration exotherm rate curve for the GFA-based alkali-activated material shows a second exothermic peak during the acceleration phase, while the curve for the group without CS continues to decline slowly. The appearance of this second exothermic peak is an important indicator of the formation of hydration products and strength development. It suggests that the hydration reaction is more intense at this stage, with Si-O, Al-O, and Ca-O bonds breaking during the induction period. During the acceleration phase, C-S-H and C-A-S-H gels rapidly form and continue to develop. Over a 168 h test period, the sample with 5% CS content accumulates a heat release of 142.82 J/g (Table 3), significantly higher than the cumulative heat release of groups with no CS or lower CS content. This indicates that higher CS content leads to a greater cumulative formation of hydration products, which results in higher strength.

**Figure 8:**
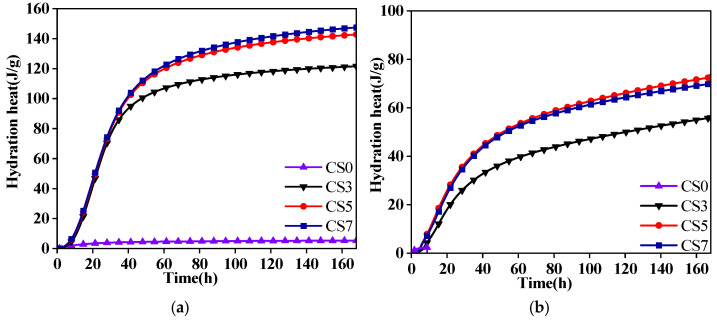
7 days cumulative heat release curves: (**a**) GGBFS; (**b**) GFA.

The cumulative heat release and hydration exotherm curves of the alkali-activated material paste over 168 h are presented in Table 3 and Figure 8. The results show that the incorporation of CS accelerates the system’s transition into the reaction phase. As CS content increases, the hydration reaction rate and cumulative heat release also increase. The hydration exotherm rate and cumulative heat release of GGBFS-based alkali-activated materials are higher than those of GFA-based alkali-activated materials. However, when the CS content exceeds 5%, both the rate of heat release and the increase in cumulative heat release start to decline.

From these results, we can conclude that the addition of CS activates the pozzolanic properties of GGBFS and FA, rapidly transitioning the system from an “inactive state” to a “reactive” one. This produces cementitious products and releases a significant amount of heat. The hydration exotherm rate and cumulative heat release of GGBFS-based alkali-activated materials are higher than those of GFA-based alkali-activated materials. This is due to the fact that FA reduces the relative amount of GGBFS in the system, which hinders the reaction between GGBFS and the alkaline activator. Additionally, FA is more difficult to activate, which results in a lower hydration exotherm and alters the cumulative heat release. The pozzolanic reaction of FA refines the pore structure, reduces permeability, and lowers the hydration heat, thereby reducing thermal cracking and improving the long-term durability of the system.

As shown in Table 3, at CS contents of 3%, 5%, and 7%, the cumulative heat release of the GGBFS group at 7 days is higher than that of the mineral powder group by 65.55 J/g, 70.11 J/g, and 77.34 J/g, respectively. These findings align with macroscopic mechanical test results, which show that the early strength of the GGBFS-based group is higher than that of the GFA-based group.

### 3.4. XRD Analysis

**Figure 9:**
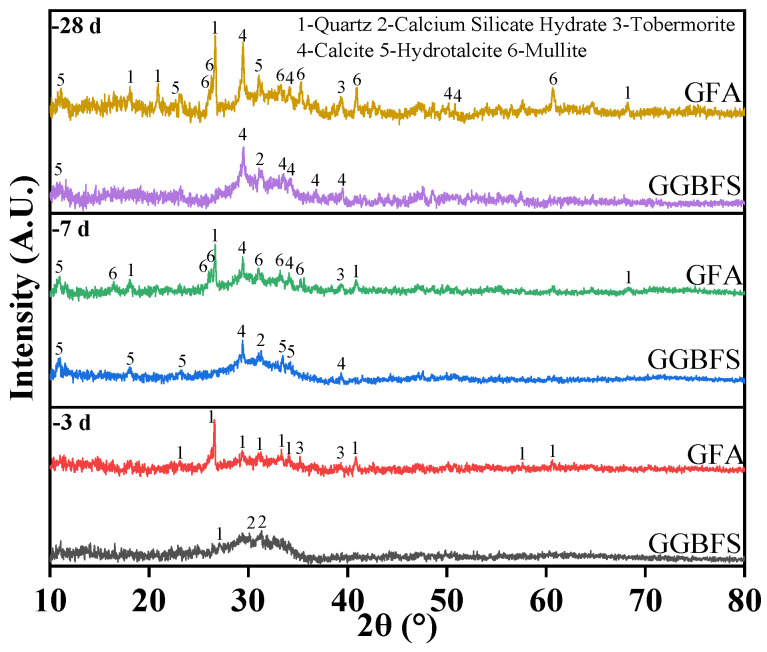
XRD spectra of hydration products of GGBFS-based and GFA-based alkali-activated materials.

The phase analysis results of the hydration products for GGBFS-based and GFA-based alkali-activated material specimens at 3 days, 7 days, and 28 days (shown in Figure 9). The results indicate the presence of six mineral phases in the samples: quartz [SiO_2_], calcium silicate hydrate [C-S-H], tobermorite [5CaO·6SiO_2_·5H_2_O], calcite [CaCO_3_], hydrotalcite [HT, 6MgO·Al_2_O_3_·CO_2_·12H_2_O], and mullite [3Al_2_O_3_·2SiO_2_]. The formation of HT is attributed to the re-polymerization of undissolved Mg^2+^ in GGBFS with the hydroxide of amorphous Al under high-pH conditions [34]. Quartz originates from the mineral phases in GGBFS and FA, while tobermorite, a crystalline calcium silicate hydrate, is derived from FA. Calcite likely forms due to the carbonation effect of CS [35].

Comparing the diffraction patterns of GGBFS-based and GFA-based alkali-activated materials, the GFA-based alkali-activated material exhibits a significant quartz peak between 20° and 30°. The GGBFS-based alkali-activated material, on the other hand, shows a diffraction peak for C-S-H gel in the 30° to 40° range, which intensifies with increasing curing time, indicating an increase in the crystallinity of the hydration products. Mullite peaks become more pronounced in the 7 days and 28 days diffraction patterns of the GFA-based alkali-activated material. Both alkali-activated material types show characteristic peaks for HT and CaCO_3_ at 7 days and 28 days, with CaCO_3_ formation attributed to curing in an open environment with exposure to CO_2_. The amount of CaCO_3_ increases as curing time progresses [36]. C-S-H gels and C-A-S-H gels are the main hydration products of CS-alkali-activated materials and are responsible for the primary strength development of these alkali-activated materials. The reaction mechanism involves Ca(OH)_2_ in CS, providing OH^−^, while FA and GGBFS release silicoaluminate components under alkaline conditions. The Ca^2+^ from CS addresses the calcium deficiency in FA, leading to the formation of C_5_S_3_A and excess calcium components, which then form C_4_AH_13_, C_2_ASH_8_, and C-S-H gel, the key products in the alkali-activation system with Ca(OH)_2_.

### 3.5. TG-DTG Analysis

**Figure 10:**
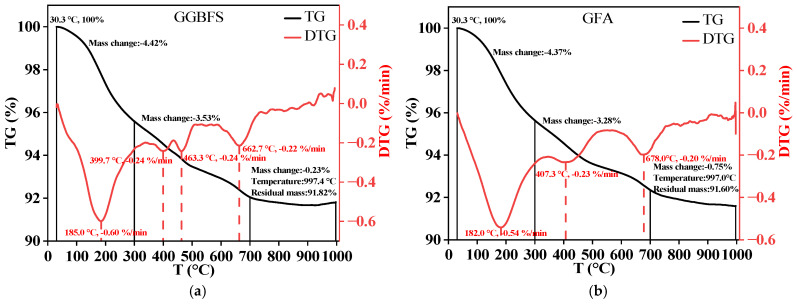
TG-DTG curves of GGBFS-based and GFA-based alkali-activated materials at 28 days. (**a**) Thermogravimetric analysis of GGBFS-based alkali-activated material; (**b**) Thermogravimetric analysis of GFA-based alkali-activated material.

**Figure 11:**
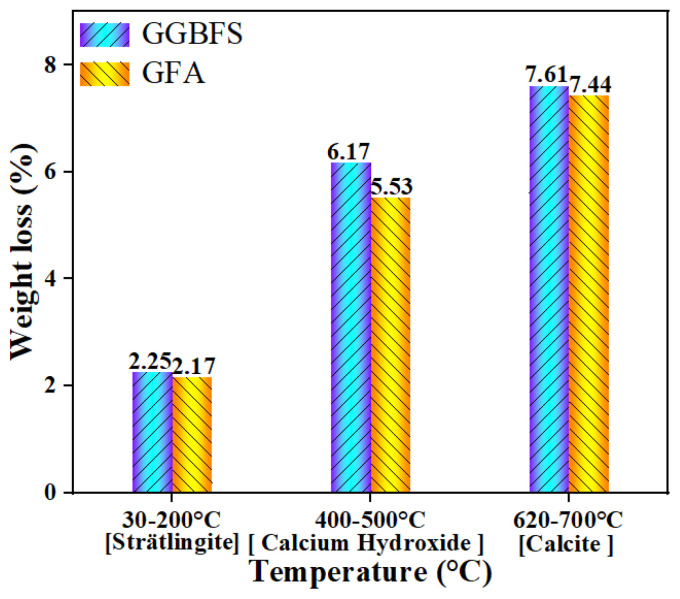
Weight loss at different temperature ranges for GGBFS-based and GFA-based alkali-activated materials.

The TG-DTG curves for GGBFS-based and GFA-based alkali-activated materials after 28 days of curing (Figure 10a,b). Both samples exhibit weight loss at temperatures of 30 °C to 200 °C, 400 °C to 500 °C, and 620 °C to 700 °C, corresponding to the decomposition of Strätlingite [14], Ca(OH)_2_ [37], and CaCO_3_ [38], respectively. The weight loss from the 30 °C to 200 °C range is 2.25% for the GGBFS-based alkali-activated material and 2.17% for the GFA-based alkali-activated material (Figure 11). This suggests that the addition of GGBFS increases the content of C-(A)-S-H gel, which contributes to the improved compressive strength of the alkali-activated material at 28 days with higher GGBFS content. The weight losses in the 400 °C to 500 °C range are 6.17% and 5.53% for the GGBFS-based and GFA-based alkali-activated materials, respectively. At 620 °C to 700 °C, the weight losses are 7.61% and 7.44%, respectively. These results align with the XRD findings at 28 days, showing that the cementitious formation and strength between the GGBFS-based and GFA-based alkali-activated materials are quite similar.

### 3.6. FTIR Analysis

**Figure 12:**
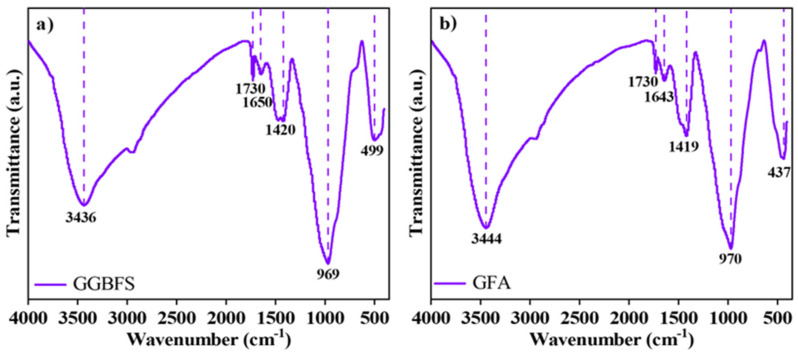
FTIR curves of (**a**) GGBFS-based alkali-activated material at 28 days, (**b**) GFA-based alkali-activated material at 28 days.

Figure 12 presents the 28 days FTIR spectra of the samples, with six distinct absorption peaks observed. Absorption peaks at approximately 3436 cm^−1^, 3444 cm^−1^, 1650 cm^−1^, and 1643 cm^−1^ correspond to the asymmetric stretching and bending vibrations of O-H bonds [39], indicating the existence of interlayer water with strong hydrogen bonding, which is mainly derived from the hydroxyl groups of C-A-S-H gel and HT. Peaks near 969 cm^−1^ and 970 cm^−1^ are assigned to the stretching vibrations of Si(Al)-O bonds [40], whereas those around 499 cm^−1^ and 437 cm^−1^ correspond to their out-of-plane and in-plane bending vibrations [41], both primarily originating from C-(A)-S-H gel. Peaks near 1420 cm^−1^ and 1419 cm^−1^ arise from the stretching vibrations of C-O bonds and out-of-plane bending vibrations of CO_3_^2−^ [40,42], derived from calcite and HT, respectively.

### 3.7. SEM-EDS Analysis

**Figure 13:**
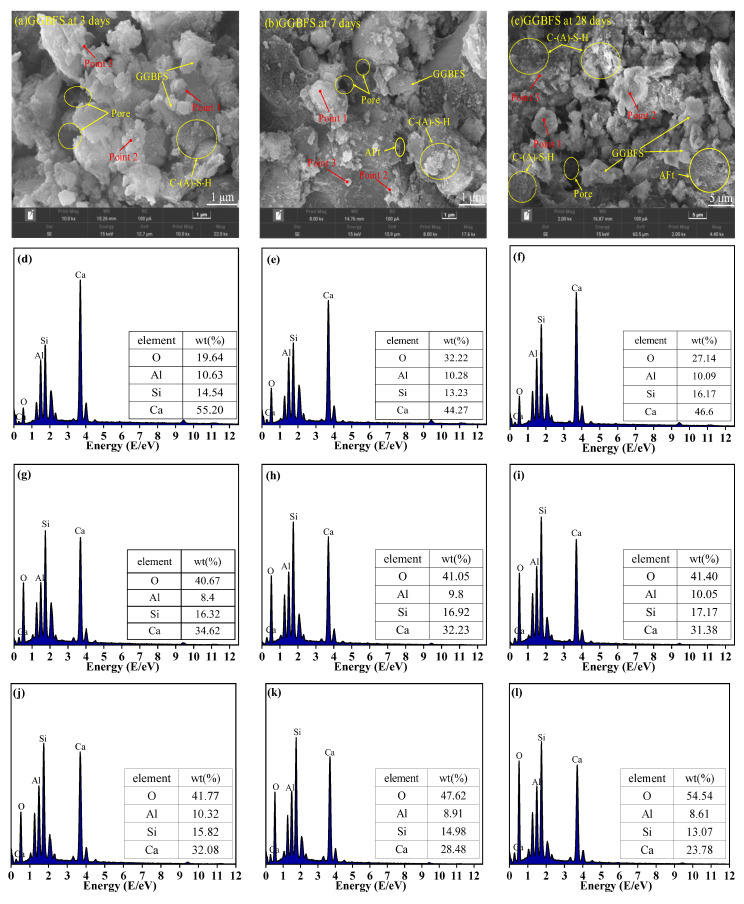
SEM images of GGBFS-based alkali-activated material at (**a**) 3 days, (**b**) 7 days and (**c**) 28 days; and EDS results (**d**) 3 days Point 1, (**e**) 3 days Point 2, (**f**) 3 days Point 3, (**g**) 7 days Point 1, (**h**) 7 days Point 2, (**i**) 7 days Point 3, (**j**) 28 days Point 1, (**k**) 28 days Point 2, (**l**) 28 days Point 3.

**Figure 14:**
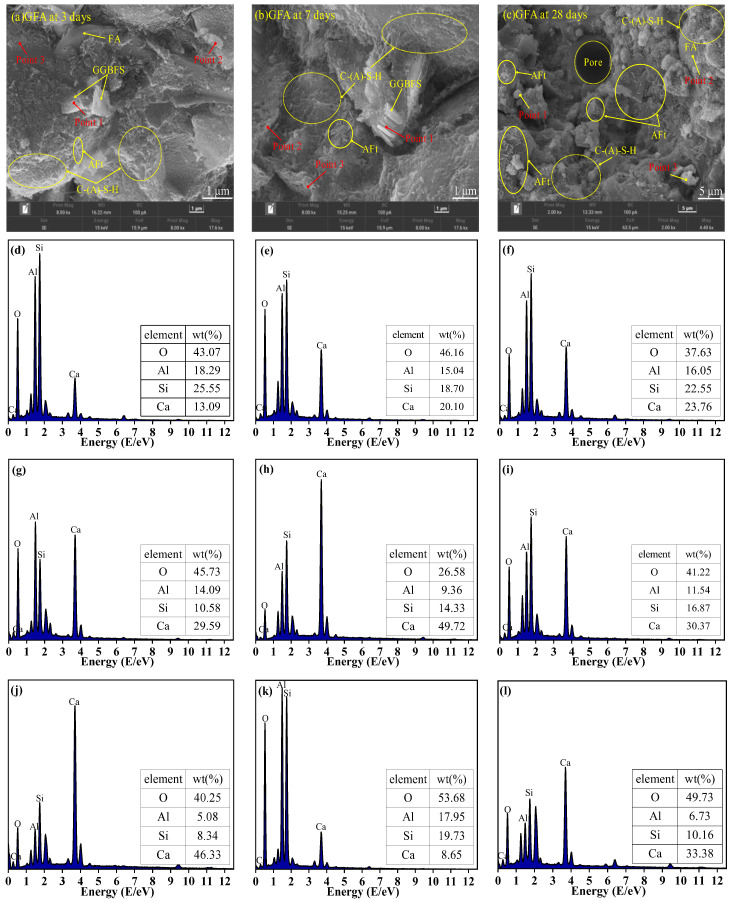
SEM images of GFA-based alkali-activated material at (**a**) 3 days, (**b**) 7 days and (**c**) 28 days; and EDS results of (**d**) 3 days Point 1, (**e**) 3 days Point 2, (**f**) 3 days Point 3, (**g**) 7 days Point 1, (**h**) 7 days Point 2, (**i**) 7 days Point 3, (**j**) 28 days Point 1, (**k**) 28 days Point 2, (**l**) 28 days Point 3.

**Figure 15:**
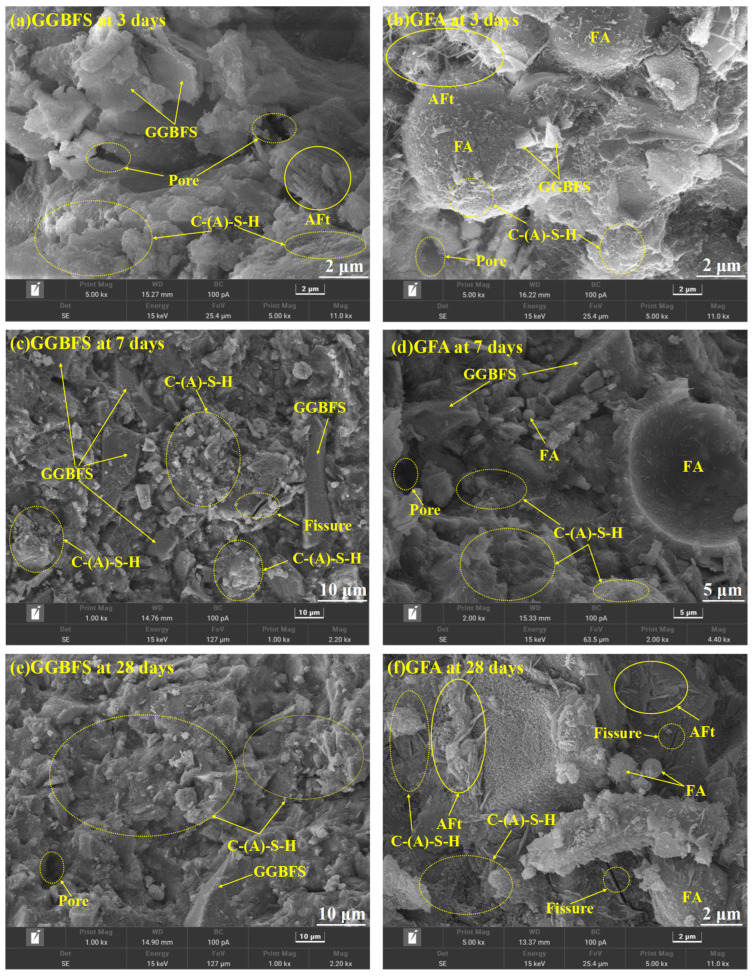
SEM images of GGBFS-based alkali-activated material (**a**,**c**,**e**) and GFA-based alkali-activated material (**b**,**d**,**f**) at 3 days, 7 days and 28 days.

Figure 13, Figure 14 and Figure 15 present the SEM micrographs of GGBFS-based and GFA-based alkali-activated materials at various curing ages. At the early hydration stage, the gel’s structure was immature with numerous visible cracks and pores. With the extension of curing age, porosity reduced while the microstructure gradually densified. Hydration products, including AFt, reticular C-S-H gels, and C-(A)-S-H gels, increased substantially [43,44], which is consistent with the FTIR findings.

Elemental analysis from EDS mapping revealed that in the first 3 days, the samples primarily contain Ca, O, and Al, indicating the presence of residual unreacted GGBFS and FA particles. At 7 days and 28 days, the samples mainly contain Ca, Si, Al, and O elements, suggesting the formation of C-S-H gels and C-A-S-H gels, which is also demonstrated by the XRD results. In the GGBFS samples, the presence of AFt is more pronounced, and the C-S-H gel appears densely packed. The pore-filling effect of AFt is thought to contribute positively to the strength development [14]. In contrast, the smoother surface and lower reactivity of FA lead to a weaker alkaline environment in the GFA group. This limits the dissolution of silicoaluminate materials, resulting in an increased number of unhydrated particles and reduced formation of silicate and aluminosilicate gels, which hinders the development of a dense microstructure and contributes to lower strength.

The Ca(OH)_2_ in CS releases substantial amounts of Ca^2+^ into the pore solution. The external Ca^2+^ reacts with Si and Al to form a gel with a high Ca/Si ratio, densifying the microstructure and increasing macroscopic strength [45] (Figure 13). FA partially replaces GGBFS, reducing the available Ca, Si, and Al in the system. As a result, the C-(A)-S-H gel in the GFA-based alkali-activated material exhibits a lower Ca/Si ratio [46]. However, due to the initially higher Al/Si ratio, Strätlingite and Al(OH)_3_ precipitate during C-(A)-S-H gel formation in the GFA group [47].

EDS results further reveal that in the early stages, a higher Ca(OH)_2_ content leads to more needle-like C-S-H gel. As hydration progresses, this needle-like C-S-H gel evolves into an amorphous form. When the amorphous C-S-H gel has a low Ca/Si ratio, silica in the C-S-H gel is gradually replaced by aluminum, forming C-(A)-S-H gel [48].

## 4. Activating Mechanism of CS

Through both macroscopic and microscopic analyses, it is clear that in GGBFS-based and GFA-based alkali-activated materials, GGBFS and FA act as precursors, providing silicoaluminate components. CS serves primarily as an alkaline activator, creating the highly alkaline environment necessary for geo-polymerization. It effectively activates both GGBFS and FA.

**Figure 16:**
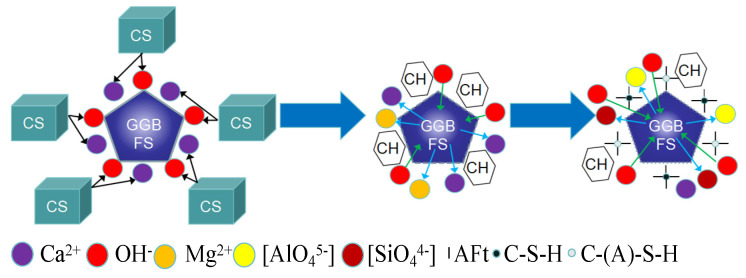
Illustration of the activation mechanism of GGBFS by CS.

GGBFS contains a significant amount of amorphous glass, which is composed of both continuous and discontinuous phases. The continuous phase is rich in calcium (the calcium-rich phase), which constitutes the majority of the GGBFS glass. In contrast, the silicon-rich phase appears as a discontinuous phase, often encapsulating the calcium-rich phase. Together, these two phases form the structural characteristics of the GGBFS glass. Figure 16 illustrates the activation mechanism of CS on GGBFS, where CS is rich in Ca(OH)_2_. In solution, there is an abundance of OH^−^ and Ca^2+^. The activation by CS creates a highly alkaline environment, disrupting the silicate protective layer on the surface of GGBFS and releasing Mg^2+^ and Ca^2+^. At this stage, with a high concentration of Ca^2+^ in the solution and before [SiO_4_]^4−^ and [AlO_4_]^5−^ have dissolved, Ca(OH)_2_ crystals begin to precipitate and accumulate around the GGBFS particles. Due to the ongoing action of the alkaline environment, the crystal structure of the GGBFS is further disrupted, causing the dissolution of [SiO_4_]^4−^ and [AlO_4_]^5−^. These dissolved ions react with the free Ca^2+^ in the solution to form C-(A)-S-H gel [49], as shown in Equations (1) and (2) [50,51]. As the GGBFS crystals break down, the concentration of [SiO_4_]^4−^ and [AlO_4_]^5−^ increases, and Ca^2+^ is progressively consumed. In this process, the precipitated Ca(OH)_2_ crystals continue to react with [SiO_4_]^4−^ and [AlO_4_]^5−^ to form more C-(A)-S-H gel, and some free Ca^2+^ will react with Al_2_O_3_ and sulfates in the GGBFS to form AFt and AFm phases (as shown in Equations (3) and (4) [50,51]).

OH^−^ + AlO^2−^ + H_2_O → [H_3_AlO_4_]^2−^ (1)

Ca^2+^ + SiO_4_^4−^ + [H_3_AlO_4_]^2−^ → CaAl_2_Si_2_O_8_·4H_2_O + OH^−^ (C-(A)-S-H) (2)

OH^−^ + AlO^2−^ + H_2_O → [Al(OH)_6_]^3−^ (3)

Ca^2+^ + [Al(OH)_6_]^3−^ + SO_4_^2−^ + H_2_O → 3CaO⋅Al_2_O_3_·3CaSO_4_·32H_2_O (AFt) (4)

4Ca^2+^ + 4Al^3+^ + 20OH^−^ + 3H_2_O → 3CaO·2Al_2_O_3_·Ca(OH)_2_·12H_2_O (5)

3Ca^2+^ + 2Si^4+^ + 14OH^−^ → 3CaO·2SiO_2_·3H_2_O + 4H_2_O (C-S-H) (6)

**Figure 17:**
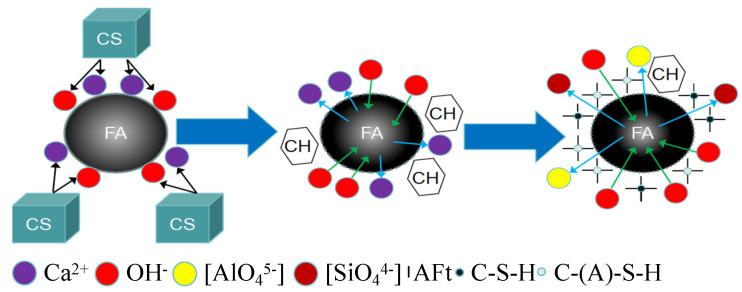
Illustration of the activation mechanism of FA by CS.

FA, which primarily consists of oxides such as SiO_2_, Al_2_O_3_, and Fe_2_O_3_, undergoes a four-stage reaction process when activated by CS to form cementitious materials (as illustrated in Figure 16 and Figure 17):

1. Dissolution Stage: Under alkaline conditions, SiO_2_ and Al_2_O_3_ in the FA particles dissolve when they react with OH^−^ ions in the alkaline solution. This breaks covalent bonds, such as Si-O and Al-O. Dissolution is a crucial part of the overall reaction process, controlling its progression.
2. Intrusion Stage: As the pH of the system increases, the acidic glassy phase of the FA is eroded by alkalis, releasing some SiO^4−^ and AlO^4−^ ions.
3. Polymerization Stage: Given the abundance of free Ca^2+^, [SiO_4_]^4−^, and [AlO_4_]^5−^ in the solution, these ions undergo polymerization to different extents. Ca^2+^ reacts with [SiO_4_]^4−^ to form C-S-H gel, while an increased Ca/Si ratio promotes the formation of C-(A)-S-H gel, leading to the coexistence of both gels in the system (as shown in Equations (5) and (6)).
4. Hardening Stage: These gels accumulate both inside and outside the FA glassy phase. As the curing process continues, the gel materials gradually dehydrate, coagulate, and harden, filling the pores of the sample. This process enhances the microstructure of the sample, making it denser and more cohesive.

## 5. Environmental Benefit Analysis

According to GB/T 51366-2019 [52], the carbon emission results of GGBFS-based alkali-activated material and GFA-based alkali-activated material are shown in Table 4. Due to the incorporation of FA, the carbon emission of the GFA-based alkali-activated material (GGBFS/FA = 70/30) decreased by 21.7% compared with the GGBFS-based alkali-activated material, while maintaining sufficient strength to meet practical requirements for structural applications. Therefore, the GFA-based alkali-activated material has better benefits.

Further analysis reveals an optimal balance point at a moderate FA replacement level (around 30–40%), where the environmental benefits are significant without causing a sharp deterioration in strength. This finding highlights the potential of tailored GGBFS-FA blends to achieve both high performance and sustainability.

## 6. Conclusions

Cement-free binders activated by CS can effectively utilize industrial solid waste to reduce carbon emissions, while their strength meets the requirements for low-intensity civil engineering applications. In this study, the performance, hydration products, and microstructure of CS-activated GGBFS-based and GFA-based alkali-activated materials were investigated. The following conclusions can be drawn:

(1) CS as an Activator: CS effectively stimulates the pozzolanic activity of GGBFS and FA. As the CS content increases, the strength of the binder improves. When the CS content is 5%, the compressive strength at 7 days of the GGBFS-based alkali-activated material is 79.7% higher compared to when the CS content is 2%. Similarly, the compressive strength at 7 days of the GFA-based alkali-activated material (with a GGBFS/FA ratio of 70/30) is 60% higher at 5% CS compared to 2% CS. The incorporation of CS accelerates the activation of the precursors, significantly increasing the hydration exotherm rate, with optimal performance achieved at a 5% CS content.
(2) Effect of GGBFS and FA Ratios: Increasing the proportion of GGBFS enhances the strength of the cementitious system but may reduce the workability of the alkali-activated material paste. On the other hand, adding FA improves the paste’s workability, reduces the hydration exotherm, and enhances durability. Therefore, with a fixed CS content, this study recommends a GGBFS to FA ratio of 7:3 for optimal performance, and at this ratio, the GFA-based alkali-activated material can reduce carbon emissions by 27.7% compared to the GGBFS-based alkali-activated material.
(3) Heat Release and Hydration: The addition of CS rapidly transforms the precursors from an “inactive” to a “reactive” state. Over a 168 h testing period, the cumulative heat release of the alkali-activated material samples with 5% CS content was 142.82 J/g and 72.71 J/g, significantly higher than the heat release from groups without CS or with lower CS content. At 5% CS, the 7 days cumulative heat release of the GGBFS-based group was 70.109 J/g, higher than that of the GFA group (with 30% FA). The incorporation of FA significantly reduces the hydration heat release, thereby improving the long-term durability and service performance of the system.
(4) Microstructural characterization: XRD, TG-DTG, FTIR and SEM-EDS results demonstrate that CS incorporation facilitates the extensive formation of AFt, C-S-H gel, C-(A)-S-H gel, and hydrotalcite-like hydrates. The formation and polymerization of the hydrates and gels underpin the strength development of the alkali-activated material system.

## Figures and Tables

**Table 1:** Chemical compositions of GGBFS, FA, and CS (%).

Materials	SiO_2_	Al_2_O_3_	Fe_2_O_3_	K_2_O	TiO_2_	MgO	CaO	Na_2_O	Loss
GGBFS	34.20	17.60	1.010	0.380	0.760	6.210	36.00	0.600	3.240
FA	47.65	34.63	6.710	1.400	1.470	0.562	5.480	0.293	1.805
CS	1.570	1.980	0.470	0.040	0.090	0.000	68.97	0.300	26.58

**Table 2:** Mixture proportion.

Group	GGBFS Content/g	FA Content/g	CS Content (Mass Fraction)/%	W/B Ratio	Water Reducing Agent Content (Mass Fraction)/%
1	1500	0	2, 3, 4 5, 6, 7, 8	0.34	0.50
2	1050	450			
3	750	750			
4	450	1050			
5	0	1500			

**Table 3:** Accumulated heat release of GGBFS-based and GFA-based alkali-activated materials at 7 days.

Group	GGBFS Accumulated Heat Release (J/g)	GFA Accumulated Heat Release (J/g)
CS0	5.31	2.38
CS3	121.59	56.04
CS5	142.82	72.71
CS7	147.52	70.18

**Table 4:** Comparison of carbon emissions between GGBFS-based alkali-activated material and GFA-based alkali-activated material.

Materials	GGBFS-Based Alkali-Activated Material (GGBFS/FA = 100/0)			GFA-Based Alkali-Activated Material (GGBFS/FA = 70/30)		
	Content (g)	Carbon Emission Coefficient (kg CO_2_e/t)	Carbon Emission (kg CO_2_e)	Content (g)	Carbon Emission Coefficient (kg CO_2_e/t)	Carbon Emission (kg CO_2_e)
GGBFS	1500	65.3	0.10	1050	65.3	0.07
FA	—	8.0	—	450	8.0	0.004
CS	75	151.1	0.01	75	151.1	0.01
Water reducing agent	7.5	1222	0.01	7.5	1222	0.01
SUM	1582.5	—	0.12	1582.5	—	0.094

## Data Availability

The datasets generated during this study are fully available within the article.
